# Are King’s College and Clichy-Villejuif criteria used for liver transplantation still appropriate? A retrospective study over a 25-year period

**DOI:** 10.1016/j.aicoj.2026.100067

**Published:** 2026-04-21

**Authors:** Clément Monet, Laurène Fusi, Yassir Aarab, Lauriane Degravi, Ines Lakbar, Mathieu Capdevila, Joris Pensier, Lucy Meunier, Jose Ursic-Bedoya, Bader Al Taweel, Fouad Belafia, Gérald Chanques, Audrey De Jong, Georges Philippe Pageaux, Samir Jaber

**Affiliations:** a Department of Anesthesia and Intensive Care Unit, Regional University Hospital of Montpellier, St-Eloi Hospital, University of Montpellier, PhyMedExp, INSERM U1046, CNRS UMR, 9214, Montpellier, CEDEX 5, France; b Montpellier University Hospital, Montpellier, France; c Department of Hepatogastroenterology and Liver Transplantation, University Hospital Center Montpellier, University of Montpellier, Montpellier, France; d Department of Surgery, Division of HBP Surgery and Transplantation, Saint-Eloi Hospital, University Hospital of Montpellier, Montpellier, France

**Keywords:** Acute liver failure, Acute liver injury, Liver transplantation, Transplantation criteria, King’s College, Clichy Villejuif

## Abstract

**Background:**

Acute liver injury (ALI) is a life-threatening condition that may require liver transplantation (LT). For over three decades, the King’s College Hospital (KCH) and Clichy-Villejuif (CV) criteria have guided LT decisions, but their relevance in the modern intensive care unit (ICU) era remains uncertain. This study aimed to assess the “real-life” incidence of LT among ICU patients with ALI fulfilling these criteria. We retrospectively analysed prospectively collected data from 2000 to 2025 in a tertiary ICU with an active LT program. All consecutive adult patients with ALI fulfilling KCH and/or CV criteria without contraindications were included. The primary endpoint was the incidence of LT. Secondary endpoints included ICU and one-year survival, organ support requirements, temporal trends and transplant-free survival according to criteria fulfilment.

**Results:**

Among 396 patients with ALI, 118 fulfilled KCH and/or CV criteria without contraindications for LT. The incidence of LT was 34% (40 out of 118). ICU and one-year survival did not significantly differ between transplanted and not transplanted patients (88% vs. 92% and 85% vs. 81%, respectively). Transplanted patients had more severe liver dysfunction, reflected by higher MELD scores driven by higher values of bilirubin and INR. They were more likely to have auto-immune hepatitis (23% vs. 1%, p < 0.01), and less likely to have acetaminophen-induced (25% vs. 46%, p = 0.03) or ischaemic ALI (2% vs. 26%, p < 0.01). Encephalopathy progression was more frequent in transplanted patients. The delay between ICU admission and LT significantly increased over the study period. Among not transplanted patients, one-year transplant-free survival exceeded 80% overall and remained high across all KCH/CV fulfilment patterns.

**Conclusion:**

In our cohort, only one-third of ALI patients fulfilling emergency LT criteria underwent transplantation. These findings suggest that historical KCH and CV criteria have limited ability to identify patients with poor prognosis without LT in the context of modern intensive care.

## Background

Acute liver injury (ALI) is defined as a potentially reversible syndrome that may result from various hepatic insults [1,2]. Its definition has evolved over time, taking into account aetiologies and interval between the onset of symptoms and the development of encephalopathy [3,4]. ALI is characterised by markers of liver damage associated with impaired liver function in patients who do not have chronic liver disease and whose illness duration is less than 26 weeks. Acute liver failure (ALF) is characterised by similar clinical features with any degree of mental status alteration (encephalopathy) [5]. ALI and ALF carry a high morbidity and mortality without liver transplantation (LT). Management is primarily supportive and focuses on the prevention or control of cerebral edema and intracranial hypertension, the correction of metabolic disturbances, and the maintenance of hemodynamic stability [5,6]. In the most severe cases, or when medical therapy fails, LT is indicated, provided there are no contraindications. ALF accounts for approximately 8% of all liver transplantations [1,3,5,7]. Survival has improved over the past decades, both in patients treated with and without LT [[8], [9], [10]]. In the modern era of growing imbalance between increasing LT indications and low graft supply, it is essential to avoid unnecessary transplants in ALF.

Over three decades ago, the King’s College Hospital (KCH) and Clichy–Villejuif (CV) criteria were developed to guide urgent LT listing [5,10]. These criteria have been widely adopted and are still recommended in contemporary guidelines. However, they were derived in an era when organ support, and peri-transplant care were less advanced than today. Several studies have since reported marked improvements in transplant-free (from 32.9% in 1998 to 61.0% in 2013 [11]) and post-transplant survival in ALF suggesting that the prognostic meaning of historical criteria may have evolved.

Importantly, most previous studies have focused on patients who were already listed for super-urgent LT, rather than on the broader population of ICU patients with ALI who fulfil transplantation criteria but may never be listed or transplanted. This real-life gap between criteria fulfilment and actual transplantation, and its consequences on outcomes, remain poorly described. We therefore conducted a long-term, single-centre cohort study of ICU patients with ALI who fulfilled KCH and/or CV criteria over a 25-year period. We hypothesised that most ALI patients meeting LT criteria (either KCH or CV) would not undergo LT. The primary objective was to describe the real-life incidence of LT in this population. Secondary objectives were to compare characteristics, clinical course, and outcomes between transplanted and not transplanted patients; to assess temporal trends in transplant practices and survival; and to evaluate the prognostic performance of KCH and CV criteria in the context of modern ICU care.

## Materials and methods

### Study design, setting, and participants

We conducted a retrospective analysis of prospectively collected data from January 2000 to March 2025, in the mixed medical–surgical ICU of a tertiary university hospital with an active liver transplantation program.

### Participants

All consecutive patients admitted to ICU were included if they met both the following inclusion criteria: (1) a diagnosis of ALI and (2) fulfilment of LT criteria (either KCH, CV, or both) regardless of waiting-list registration. Patients were excluded if they: (1) had ALI without meeting LT criteria, (2) had evidence of cirrhosis, (3) were admitted after hepatic resection surgery, or (4) had absolute contraindications for LT (e.g. irreversible multiorgan failure, active malignancy, or major psychiatric condition precluding post-transplant adherence) as determined by a multidisciplinary transplant team. ALI was defined by the presence of: a) elevation of transaminases, b) INR ≥ 1.5 or PT < 50%, c) acute illness onset <26 weeks, d) no evidence of cirrhosis [4]. Included patients were classified into two predefined groups: the transplanted group, including all ALI patients fulfilling KCH and/or CV criteria who ultimately underwent LT; and the not transplanted group, including ALI patients fulfilling KCH and/or CV criteria but who were not transplanted, regardless of whether they were listed for LT.

### Liver transplantation criteria

KCH and CV criteria were defined according to the original publications and current guidelines (Supplementary Table S1) [[12], [13], [14]]. As indicated in these criteria, severe hepatic encephalopathy is required for the Clichy–Villejuif criteria but not systematically for the King’s College criteria. For analysis, we classified patients as fulfilling KCH, CV, both, or neither. Encephalopathy was graded according to the West Haven scale. In patients receiving sedatives or with suspected toxic co-ingestions (e.g. benzodiazepines, antidepressants), the potential for confounding was explicitly recognised and addressed in the limitations.

### Clinical management and transplant decision process

All patients were managed according to evolving local protocols and international guidelines, including identification and treatment of the underlying cause, prevention and management of cerebral oedema, hemodynamic optimisation, organ support and treatment of infections. Extracorporeal liver support (e.g. MARS®) was available throughout most of the study period. Decisions to list for emergency LT and to proceed to transplantation were taken by a multidisciplinary team including hepatologists, transplant surgeons, anaesthesiologists and intensivists.

### Ethical approval

The study was approved by the Institutional Review Board of Montpellier University Hospital (2019_IRB-MTP_05-25). The need for informed consent was waived due to the retrospective nature of the study.

### Data collection

Clinical data were prospectively recorded and retrospectively reviewed from the ICU database. Variables were selected based on prior hepatology and critical care literature. Data included demographics, comorbidities, aetiology (e.g. acetaminophen, ischaemia, viral, autoimmune, drug-induced, heat stroke, Amanita, Budd–Chiari, indeterminate), admission severity scores, laboratory values at admission and at their worst (pre-transplant for the transplanted group, and during ICU stay for the not transplanted group), neurological status, organ support therapies, LT listing and transplantation, ICU length of stay, and vital status at ICU discharge and one year. Hepatic encephalopathy was graded from 1 to 4 according to the West Haven criteria [15].

### Endpoints

The primary endpoint was the incidence of LT among ICU patients with ALI fulfilling KCH and/or CV criteria and without absolute contraindications. Secondary endpoints included ICU and one-year survival, ICU length of stay, use of organ supports (vasopressors, mechanical ventilation, and renal replacement therapy), ventilator-, vasopressor- and dialysis-free days at day 28 and temporal trends in LT incidence and survival over the 25-year period. In additional exploratory analyses, we examined the association between KCH and CV criteria and one-year survival and described outcomes according to the pattern of criteria fulfilment (KCH only, CV only, both).

### Statistical analysis

Continuous variables are reported as mean (±standard deviation (SD)) or median [interquartile range, 25–75%] and compared using the Student-t test or Wilcoxon rank-sum test, as appropriate. Categorical variables are expressed as numbers (percentages) and compared using the Chi-square test or Fisher’s exact test, as appropriate. In case of missing values, the number of missing values was clearly stated for each variable, and no replacement method was used. A descriptive analysis was performed overall and in transplanted patients (transplanted group) and in patients who were not (not transplanted group). Baseline characteristics, ICU course and outcomes were compared between transplanted and not transplanted patients to describe factors associated with liver transplantation decisions. Survival curves were set up until one year using the Kaplan–Meier method and compared with the log-rank test. For comparisons of one-year transplant-free survival according to criteria fulfilment, risk ratios (RR), risk differences (RD), and exact p-values were calculated using Fisher’s exact test. Finally, to assess the potential impact of evolving clinical practices, we conducted a trend analysis across the 25-year period with the Cochran–Armitage test or the Mann-Kendall test when appropriate. In addition, a predefined era-based analysis was performed by dividing the cohort into three time intervals with approximately equal sample size to compare transplantation practices, survival outcomes, and baseline characteristics across periods.

Statistical significance was considered at *p* < 0.05; p values were two-tailed. Statistical analysis was performed with EasyMedStat (version 3.42; www.easymedstat.com). Because of sample size constraints and the limited number of events, we did not perform multivariable modelling to identify independent predictors of LT or survival. The study was reported according to the Strengthening the Reporting of Observational Studies in Epidemiology (STROBE) reporting guideline statement.

## Results

### Study population

**Fig. 1:**
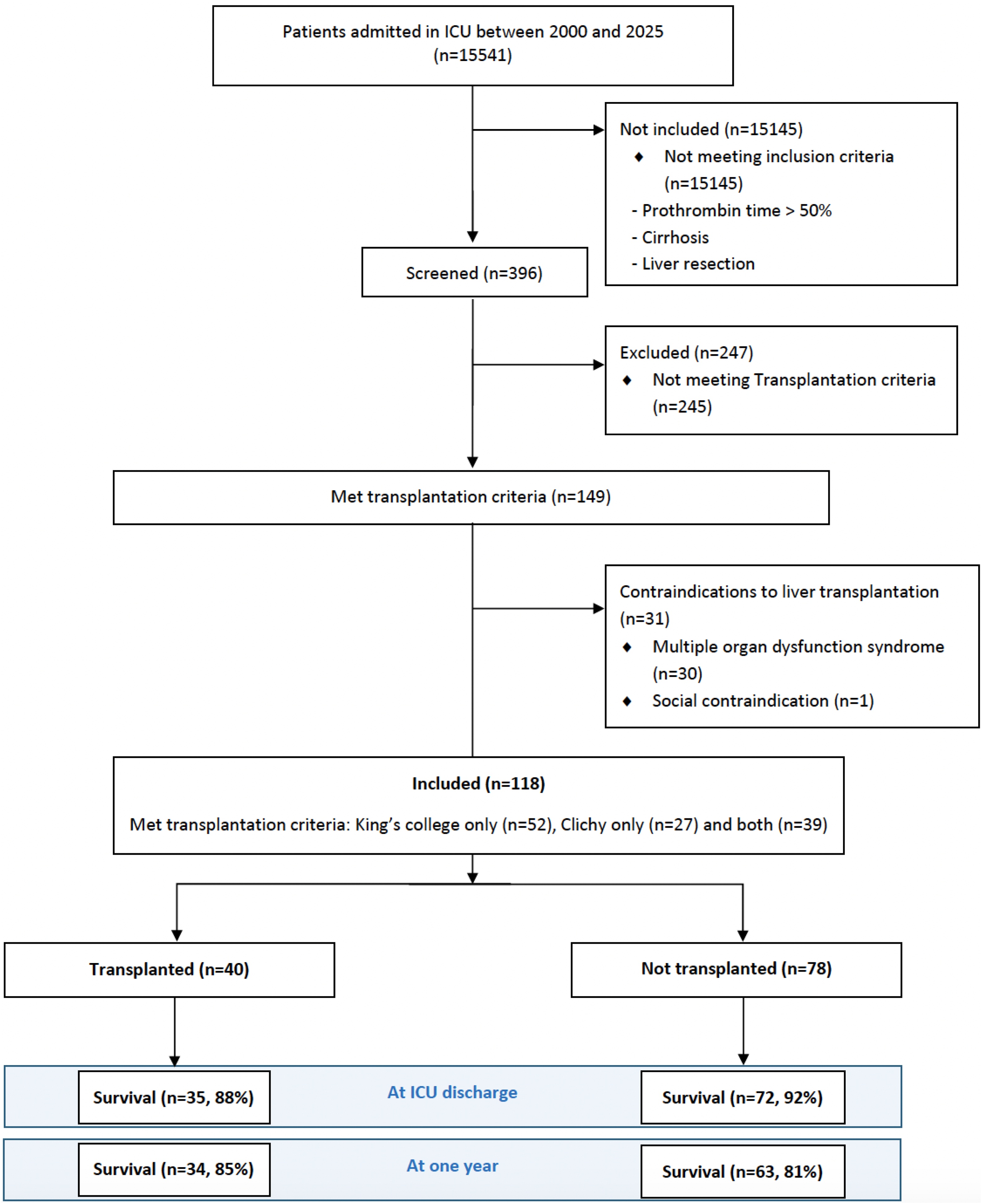
Flow chart and survival rates.

Over the 25-year period, 396 ICU patients met the diagnosis criteria for ALI. Among them, 149 fulfilled at least one of the KCH or CV criteria for emergency LT. Thirty-one patients had absolute contraindications for transplantation (refractory multiorgan failure (*n* = 30) or major psychiatric issues (*n* = 1)) and were excluded, leaving 118 eligible patients for the main cohort (Fig. 1). Among these patients, 40 (34%) underwent LT and 78 (66%) did not. Baseline characteristics are presented in Table 1. The main aetiology was acetaminophen-induced ALI (46/118, 39%). At ICU admission, 56/118 (47%) had no encephalopathy, 22/118 (19%) had grade 1–2, and 40/118 (34%) had grade 3–4 encephalopathy. Mechanical ventilation and vasopressor were required in 32/118 (27%) and 25/118 (21%) patients respectively.

**Table 1:** Patients characteristics at admission.

	Overall (*n* = 118)	Transplanted (*n* = 40)	Not Transplanted (*n* = 78)	*p-value*
Gender, female	74 (62)	26 (65)	47 (60)	0.76
Age (years)	50 (±16)	46 (±15)	48 (±16)	0.45
BMI (kg/m^2^)	24 (±5)	24 (±5)	24 (±4)	0.60
Etiology				
Acetaminophen	46 (39)	10 (25)	36 (46)	0.03
Viral hepatitis	8 (7)	5 (13)	3 (4)	0.12
HSV	5 (4)	3 (8)	2 (3)	0.33
Ischaemic	21 (18)	1 (2)	20 (26)	<0.01
Drug induced	8 (7)	1 (2)	7 (9)	0.27
Auto immune	10 (8)	9 (23)	1 (1)	<0.01
Unknown	11 (9)	9 (23)	2 (3)	<0.01
Heat shock	5 (4)	1 (2)	4 (5)	0.66
Amanita intoxication	2 (2)	1 (2)	1 (1)	0.99
Budd Chiari	2 (2)	0 (0)	2 (3)	0.55
Encephalopathy				
No	56 (47)	23 (58)	33 (42)	0.13
Grade 1 and 2	22 (19)	11 (28)	11 (14)	0.09
Grade 3 and 4	40 (34)	6 (15)	34 (44)	<0.01
Mechanical ventilation	34 (28)	4 (10)	30 (44)	<0.01
Dialysis	4 (3)	1 (2)	3 (4)	0.99
Use of vasopressors	25 (21)	2 (5)	23 (29)	<0.01
SAPS II score	50 (±20)	51 (±18)	49 (±22)	0.68
SOFA score	11 (±6)	12 (±5)	10 (±6)	0.11
MELD score	28.1 (±10.6)	31.3 (±7.9)	26.9 (±11.6)	0.006
Total Bilirubin (μmol/L)	144 (±175)	284 (±221)	71 (±78)	<0.01
Prothrombin time (%)	24 (±16)	19 (±11)	24 (±16)	<0.01
Creatinine (μmol/L)	144 (±125)	117 (±117)	159 (±129)	0.10
Platelets (G/L)	161 (±109)	183 (±129)	146 (±96)	0.14
ALT (UI/L)	3387 (±3224)	2732 (±3144)	3911 (±3266)	0.09
AST (UI/L)	4421 (±4595)	3464 (±4185)	5276 (±5038)	0.06
Factor V (%)	26 (±24)	26 (±18)	25 (±22)	0.82
INR	4.0 (±1.7)	4.5 (±1.7)	3.8 (±1.6)	0.02
HCO3- (mmol/L)	20.7 (±6.5)	20.3 (±7.2)	20.5 (±6.1)	0.79
Lactate (mmol/L)	5.6 (±4.8)	5.9 (±6.0)	5.8 (±4.4)	0.91
pH	7.3 (±0.04)	7.4 (±0.02)	7.3 (±0.05)	0.07

Quantitative data are presented as mean (±standard deviation), and qualitative as number (percentage). p-value is the result of a comparison between transplanted and not transplanted group.

Abbreviations: BMI: body mass index, HSV: herpes simplex virus, SOFA: sepsis related organ failure assessment, SAPS II: Simplified Acute Physiology Score, ALT Alanine aminotransferase, AST: Aspartate aminotransferase, INR: international normalized ratio, MELD: model for end stage liver disease.

### Main endpoint

Out of 118 ALI patients meeting LT criteria, 40 (34%) underwent liver transplantation (Fig. 1).

### Secondary endpoints

#### Comparison between groups

At ICU admission, transplanted and not transplanted patients had similar age, sex and body mass index (Table 1). Patients in the transplanted group were less likely to require mechanical ventilation (10% vs. 44%, p < 0.01) and vasopressors (5% vs. 29%, p < 0.01), at admission. Autoimmune hepatitis was more common in the transplanted group (23% vs. 1%, p < 0.01), whereas acetaminophen-induced (25% vs. 46%, p = 0.03) and ischaemic ALI (2% vs. 26%, p < 0.01) were more frequent in the not transplanted group.

At admission, MELD scores were significantly higher in the transplanted group, largely driven by higher bilirubin and INR values (Table 1). During ICU stay, worst MELD scores were significantly higher in the transplanted group, reflecting higher bilirubin and INR values (Supplementary Table S2). During ICU stay, there was no significant difference in the proportion of patients requiring mechanical ventilation, vasopressors, or renal replacement therapy (Supplementary Table S3). Similarly, the duration of mechanical ventilation did not differ significantly between groups.

In the transplanted group, the proportion of patients without encephalopathy was 58% at baseline vs. 13% before LT (p < 0.001). In contrast, the severity of encephalopathy remained stable in the not transplanted group (Supplementary Fig. S1). Evolution of biological parameters from admission to their worst values is shown in Supplementary Fig. S2. Significant deterioration was observed in the transplanted group for INR, creatinine, lactate, factor V, and platelet count.

#### Survival and ICU course

**Fig. 2:**
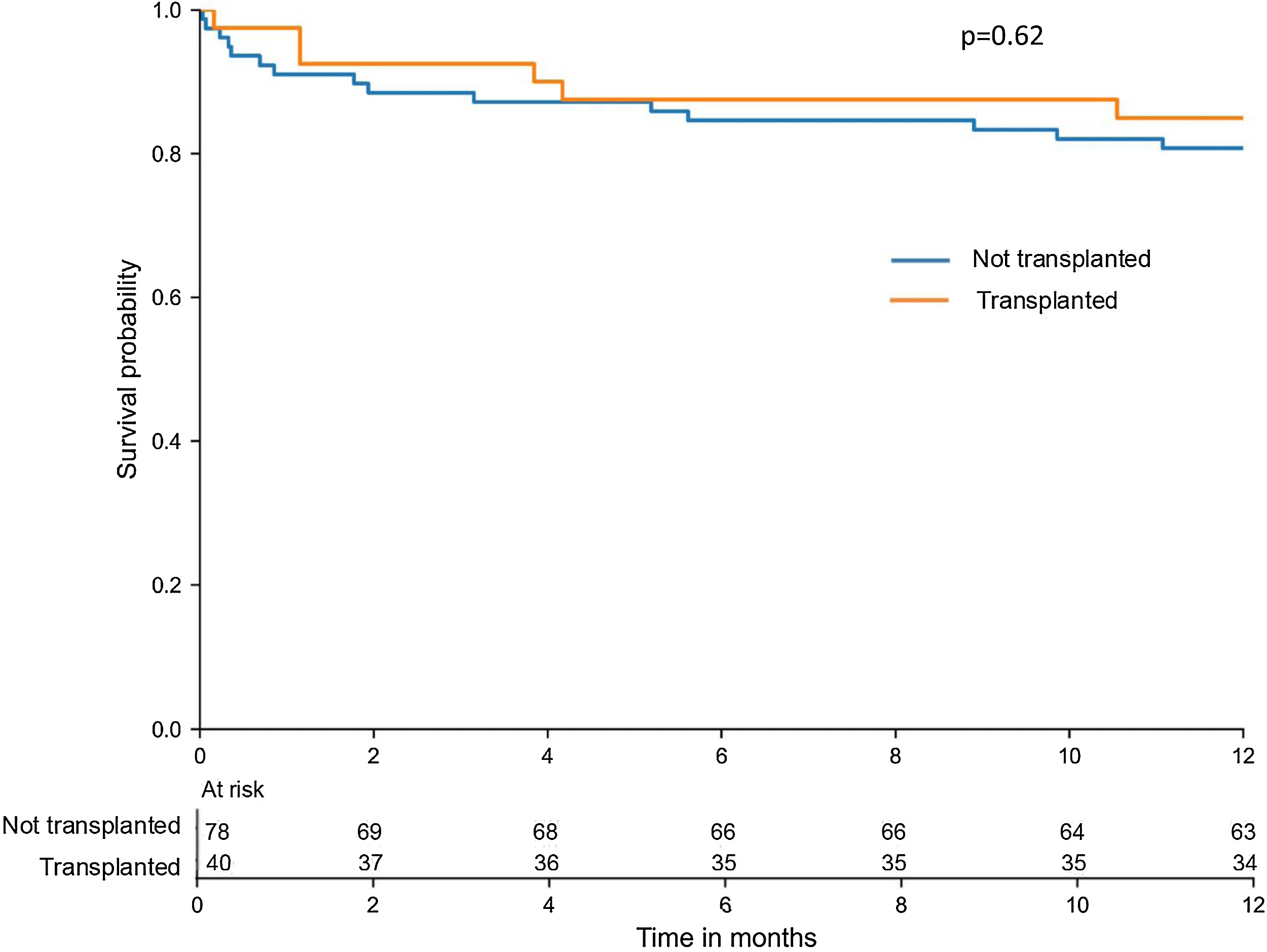
Kaplan Meier survival curve one year after admission in the ICU in both transplanted and not transplanted patients.

There was no significant difference in ICU and one-year survival rates between transplanted and not transplanted patients (88% vs 92%, p = 0.51 and 85% vs 81%, p = 0.62 respectively) (Fig. 1, Fig. 2). The characteristics of the 21 deceased patients are reported in the Supplementary Table S4. Transplanted patients had a significantly longer ICU length of stay (24.9 (±24.3) days vs 11.0 (±13.2) days, p = 0.001) and fewer ventilatory-free and dialysis-free days at day 28 (Table 2).

**Table 2:** Outcomes.

	Transplanted (*n* = 40)	Not Transplanted (*n* = 78)	*p-value*
Length of stay, days	24.9 (±24.3)	11.0 (±13.2)	*<*0.001
Ventilatory free days at d28, days	20.8 (±8.2)	23.3 (±7.1)	0.01
Dialysis free days at d28, days	23.1 (±8.2)	25.9 (±5.4)	0.04
Vasopressor free days at d28, days	25.2 (±6.4)	25.7 (±5.6)	0.68
Multiple organ dysfunction syndrome	28 (70)	42 (54)	0.13
ARDS	5 (13)	9 (12)	0.53
Mortality in ICU	5 (13)	6 (8)	0.29
Listed for transplantation	40 (100)	5 (6)	<0.001

Quantitative data are presented as mean (±standard deviation), qualitative date are presented as number (percentage).

Abbreviations: ARDS: acute respiratory distress syndrome, ICU :intensive care unit, d28: day 28 after admission.

#### Temporal trends and transplantation details

**Fig. 3:**
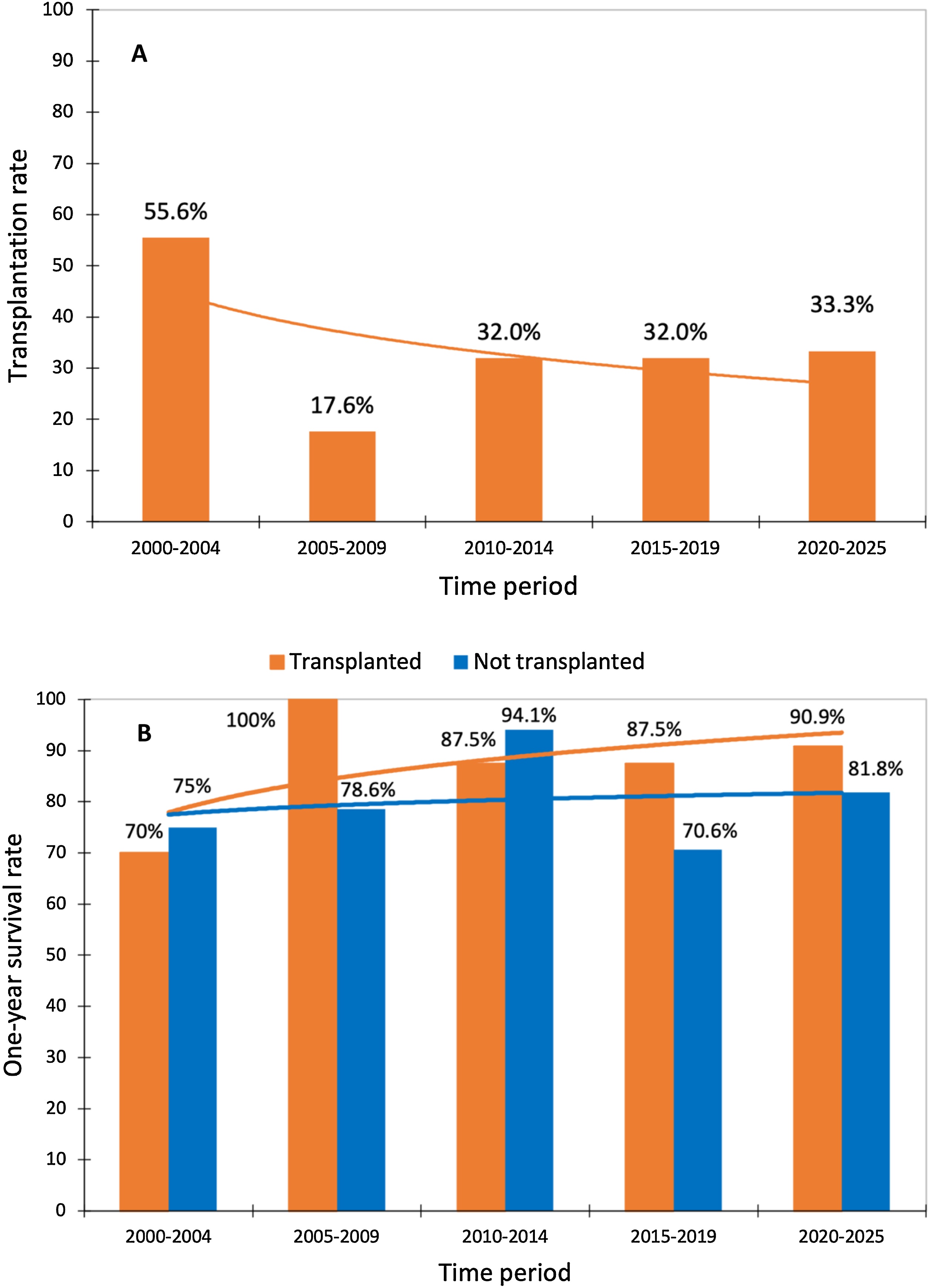
A: Evolution of transplantation rate over the study period. *p* = 0.22 per the Cochran-Armitage test for trends over time. B: Evolution of survival rate over the study period in transplanted and not transplanted patients. *p* = 0.45 and *p* = 0.89 respectively by the Cochran-Armitage test for trends over time.

Over the 25-year study period, the incidence of LT among eligible patients did not change significantly (p = 0.22) (Fig. 3A). One-year survival in transplanted and not transplanted patients also remained stable over time (Fig. 3B). To account for the long study period, we performed an additional era-based analysis dividing the cohort into three predefined intervals (2000–2010, 2011–2018, and 2019–2025). Listing rates, transplantation rates, and one-year survival remained broadly stable across eras (Supplementary Table S5). Baseline characteristics and organ support strategies were generally comparable across periods, although severe hepatic encephalopathy at admission was more frequent in later eras (Supplementary Table S6). The mean delay between ICU admission and LT was 97 (±90) hours. This delay increased significantly over time: 62 (±58) hours in 2000–2010 versus 122 (±102) hours in 2011–2025 (p = 0.05).

Out of 45 listed patients, 40 (88%) underwent LT. Among the five patients who were listed but not transplanted (Table 2), four recovered without LT (three acetaminophen-related and one drug-induced ALI). One patient, with heatstroke-related ALI, developed cerebral oedema and died in the ICU.

### Criteria fulfilment patterns and prognostic performance

Among the 118 eligible patients, 52 (44.1%) fulfilled King’s College Hospital (KCH) criteria only, 27 (22.9%) fulfilled Clichy–Villejuif (CV) criteria only, and 39 (33.1%) fulfilled both criteria. Liver transplantation was performed in 15/52 (28.8%) patients fulfilling KCH criteria only, 6/27 (22.2%) fulfilling CV criteria only, and 19/39 (48.7%) fulfilling both criteria. Among not transplanted patients, one-year transplant-free survival was 73.0% (27/37) in those fulfilling KCH criteria only, 95.2% (20/21) in those fulfilling CV criteria only, and 85.0% (17/20) in those fulfilling both criteria. When analysed by individual criteria, one-year survival was 77.2% (44/57) when KCH criteria were fulfilled versus 95.2% (20/21) when they were not (RR 0.81, 95% CI 0.69–0.95; p = 0.094). Conversely, one-year survival was 90.2% (37/41) when CV criteria were fulfilled versus 73.0% (27/37) when they were not (RR 1.24, 95% CI 1.00–1.54; p = 0.066).

## Discussion

In this 25-year single-centre ICU cohort of patients with ALI fulfilling emergency liver transplantation criteria, only one third ultimately underwent transplantation, while transplant-free survival was high across all criteria patterns. Notably, more than 80% of not transplanted patients were alive at one year. Together, these findings suggest that historical transplantation criteria have limited prognostic discrimination in contemporary intensive care practice and may not reliably identify patients with poor transplant-free outcomes [16].

While our study does not allow for the development of a new prognostic model, it underscores the need for a prospective, multicenter study with an independent validation cohort to establish more sensitive and specific selection criteria. Indeed, in a relative organ shortage context, graft allocation optimization is primordial. Importantly, this work provides an intensivist’s perspective on ALI management in the ICU and reflects real-life decision-making.

### Aetiology and decision-making

Approximately 40% of patients in our cohort had acetaminophen-induced ALI, yet this subgroup represented only 25% of those who were transplanted. Conversely, autoimmune hepatitis and ALI of unknown origin were significantly more represented among transplanted patients. These results are consistent with prior findings showing that non-acetaminophen and non-ischaemic aetiologies are often associated with less favourable outcomes and are more likely to lead to transplantation [17]. Despite being a leading indication for superurgent LT in France since 2002 [18], only 10 out of 46 (21.7%) acetaminophen-induced ALI patients in our study were actually transplanted. In contrast with Ichai et al. [10], where 26.9% of listed acetaminophen cases died or left list for worsening condition and 45.5% improved spontaneously, all not transplanted patients in our cohort survived. On the other hand, 90% of autoimmune hepatitis were transplanted. Taken together, these observations suggest that transplantation decisions were not based solely on the fulfilment of KCH or CV criteria but also incorporated aetiology and dynamic clinical evolution during ICU stay. In our cohort, extracorporeal liver support with MARS® was used in only a small proportion of patients and did not differ significantly between transplanted and not transplanted patients.

### Hepatic encephalopathy

The presence of an altered level of consciousness resulting from hepatic encephalopathy remains a key prognostic feature of ALF [3,5]. As expected, transplanted patients had a higher rate of neurological degradation in our cohort (Supplementary Fig. S1). However, five patients were transplanted without any clinical signs of hepatic encephalopathy. Mostly in cases of subacute hepatitis with poor biological evolution (mean bilirubin of 500 μmol/L), emphasizing that encephalopathy is not always required to justify LT. Finally, in our cohort the worsening of hepatic encephalopathy seemed to be the main difference between transplanted and not-transplanted patients.

### Biological variables and scoring systems

Transplanted patients had significantly higher MELD scores as well as higher values of its biological components (bilirubin and INR) at admission and during their ICU course. In our cohort, bilirubin appeared to be one of the most discriminant biological markers separating transplanted and not transplanted patients, in line with previous findings [10,19]. Transaminases, however, were lower in patients that were not transplanted, confirming their limited value in assessing liver function severity [20].

Our data also suggest that the use of dynamic markers, such as worsening coagulation parameters (particularly INR, Factor V), may help guiding transplant decisions [21]. While the model for end-stage liver disease (MELD) score has shown some predictive value [22,23], recent evidence suggests that dynamic models or organ dysfunction scores such as SOFA (sequential organ failure assessment) may offer better sensitivity and negative predictive value [24]. In our cohort, SOFA did not distinguish the groups, likely due to a high prevalence of multiorgan failure in both arms. However, MELD score was significantly higher at admission and during ICU stay in the transplanted group.

### Improved critical care and evolving practices

The increased delay between ICU admission and LT over time, from 62 to 122 h, reflects an evolving strategy of watchful waiting. This may be explained by improved overall ICU care over the past two decades benefiting ALI patients admitted in ICU. Advances such as lung-protective ventilation [25], extracorporeal liver support systems [26], refined sedation protocols [27], metabolic management [28], and optimized hemodynamic strategies [29] have likely contributed to improved transplant-free survival. Better diagnosis and management of infections, including invasive fungal infections [30,31], and enhanced use of non-invasive ventilation and weaning protocols [32,33], may also have influenced clinical decisions and outcomes. These improvements have probably helped in a more conservative, individualized approach to transplantation in ALI, allowing careful observation over immediate listing in select patients. This evolving strategy may, in turn, contribute to a more conservative selection of transplant candidates and aligns with the broader goal of optimizing graft allocation in a context of relative organ shortage.

### Strengths and limitations

This study has several strengths. First, it represents an original long-term analysis of ICU patients with ALI meeting KCH and/or CV criteria, regardless of listing status, over a 25-year period. Second, it provides real-world insight into evolving practices in critical care and transplantation. Third, the rigorous data collection from a single tertiary care center ensures consistency in decision-making. Unlike most studies who enrolled only ALF patients, we chose to include all patients with ALI, which can be seen as a limitation since in literature mostly ALF are transplanted, but also a strength since encephalopathy is not required to meet the KCH criteria. However, several limitations must be acknowledged. The single-center and retrospective design may limit the generalizability of our findings and introduces a risk of selection bias. Similar survival between transplanted and not transplanted patients may partly reflect effective clinical selection of patients most likely to benefit from transplantation. Because the study spans a 25-year period, temporal changes in ICU management could have influenced the results. However, an era-based analysis showed broadly stable survival and transplant-free survival across periods. Additionally, our study cannot establish causality or propose new prognostic tools. Nonetheless, the observed discrepancies between eligibility and actual transplantation highlight a potential mismatch between criteria and clinical judgment. We did not perform multivariate analysis to predict need to transplantation and/or survival due to restricted sample size which limited the validity of the analysis. Moreover, this study did not specifically address technical evolutions in liver transplantation itself, such as graft preservation strategies or donor management, as the primary objective was to evaluate the clinical decision-making process leading to transplantation. Finally, although our findings provide insight into real-life decision-making in a tertiary center with an active transplantation program, they require confirmation in independent multicenter cohorts before any change in clinical practice can be considered.

### Future directions

In 2000 Shakil et al. stated that validation studies have shown that, transplant-free survival in patients who meet the KCH criteria was consistently lower than 15% [34]. Yet in our cohort, among 78 patients meeting LT criteria transplant-free survival rates were 94% in ICU and 84% at one year. Since their development over three decades ago, the KCH and CV criteria have been widely used to guide urgent liver transplantation. Many static and dynamic variables have been proposed to improve or replace these criteria. This rather long list includes, among others: age, aetiology, stage of encephalopathy, serum bilirubin, serum phosphate, alfa-fetoprotein, factor VII [35] liver volume [36]. Our results call into question the continued appropriateness of current criteria in guiding LT decisions. However, this study did not allow us to establish new and better criteria. Future research should aim to validate these observations in large prospective multicenter cohorts and to develop updated prognostic tools integrating modern intensive care management.

## Conclusion

In this single center study of ICU patients with ALI, only one third of patients meeting transplantation criteria were eventually transplanted. Despite fulfilling either KCH or CV criteria, the majority were not transplanted, yet demonstrated high short- and long-term survival. These findings suggest that, although KCH and Clichy–Villejuif criteria remain valuable tools to identify patients at high risk, their prognostic discrimination may be limited in contemporary ICU practice and may not fully reflect real-world clinical decision-making. Larger prospective multicenter studies are needed to confirm these observations and to define updated criteria reflecting modern advances in critical care.

## CRediT authorship contribution statement

SJ, CM, LF, and GPP designed the study. LF and CM collected the data. LF, LD, JP, SJ, YA, IL, MC, LM, CM, and YA analysed the data. LF, LG, JP, CM, IL, MC, YA, LM, ADJ, GPP, JUB, and SJ were involved in the data interpretation. LF, LD, YA, LM, CM and SJ wrote the manuscript. CM, LF, YA, LD, IL, MC, JP, LM, JUB, BAT, ADJ, GPP and SJ revised and approved the manuscript.

## Ethics approval and consent to participate

We obtained approval from the Montpellier University Hospital ethics committee for the Forever project on May 29, 2019 (Comité Local d’Ethique Recherche, agreement number: 2019_IRB-MTP_05-25). The requirement for informed consent was waived, and the opportunity to opt out of the study was provided. The study has been performed in accordance with the Helsinki declaration of 1975.

## Consent for publication

Not applicable.

## Funding

The funding source was the Montpellier University Hospital (France). The funder had no role in study design, data collection, analysis, interpretation, or manuscipt preparation.

## Availability of data and materials

Research data and other material (e.g., study protocol and statistical analysis plan) will be made available to the scientific community, immediately on publication, with as few restrictions as possible. All requests should be submitted to the corresponding author who will review with the other investigators for consideration. A data use agreement will be required before the release of participant data and institutional review board approval as appropriate.

## Declaration of competing interest

The authors declare the following financial interests/personal relationships which may be considered as potential competing interests:

Samir Jaber reports a relationship with Dräger Medical GmbH that includes: consulting or advisory. Samir Jaber reports a relationship with Medtronic Inc that includes: consulting or advisory. Samir Jaber reports a relationship with Mindray Medical Australia Pty Ltd that includes: consulting or advisory. Samir Jaber reports a relationship with Fresenius Medical Care AG that includes: consulting or advisory. Samir Jaber reports a relationship with Baxter International Inc that includes: consulting or advisory. Samir Jaber reports a relationship with Fisher & Paykel Healthcare Limited that includes: consulting or advisory. Audrey De Jong reports a relationship with Medtronic Inc that includes: speaking and lecture fees. Audrey De Jong reports a relationship with Dräger Medical GmbH that includes: speaking and lecture fees. Audrey De Jong reports a relationship with Fisher & Paykel Healthcare Limited that includes: speaking and lecture fees. Clement Monet reports a relationship with Medtronic Inc that includes: speaking and lecture fees. Given their role as Associate Editors, Samir Jaber and Ines Lakbar had no involvement in the peer review of this article and had no access to information regarding its peer review. Full responsibility for the editorial process for this article was delegated to another journal editor.

## Footnotes

Appendix A Supplementary data associated with this article can be found, in the online version, at http://dx.doi.org/10.1016/j.aicoj.2026.100067.
