# Supplementary material for: Are King’s College and Clichy-Villejuif criteria used for liver transplantation still appropriate? A retrospective study over a 25-year period
Source: Ann Intensive Care. 2026 Apr 21;16:100067. doi: 10.1016/j.aicoj.2026.100067 (PMC13123610; doi:10.1016/j.aicoj.2026.100067)
Supplement: Supplementary file 1 [file mmc1.docx]

**SUPPLEMENTARY APPENDIX**

**Supplementary Table S1: definition of the transplantation criteria**

| King’s college criteria |
| --- |
| ALF due to acetaminophen poisoning   - Arterial pH <7.3 after resuscitation - Lactate > 3mmol/l or - The following 3 criteria   - Hepatic encephalopathy > grade 3   - Serum creatinine > 300µmol/l   - INR > 6.5   ALF not due to acetaminophen poisoning   - INR > 6.5 or - 3 out of 4 following criteria   - Age < 10 years or > 40 years   - Interval jaundice-encephalopathy > 7 days   - Bilirubin > 300µmol/l   - INR > 3.5 |
| Clichy Villejuif criteria |
| - Hepatic encephalopathy stage 3 or 4 - Factor V < 20% if age < 30 year   Or   - Factor V < 30 % if age > 30 years |

**Supplementary Table S2. Comparison of worst biological variables between the transplanted and non-transplanted group.**

|  | **Transplanted (n= 40)** | **Not Transplanted (n=78)** | ***p-value*** |  |
| --- | --- | --- | --- | --- |
| Total Bilirubin (µmol/l) | 402 (±200) | 150 (±144) | *<.001* |  |
| Prothrombin time (%) | 11 (±5) | 18 (±9) | *<.001* |  |
| Creatinine (µmol/l) | 198 (±152) | 217 (±183) | *0.83* |  |
| Platelets (G/l) | 74 (±79) | 86 (±63) | *0.39* |  |
| ALT (UI/l) | 3686 (±3864) | 5083 (±3347) | *0.69* |  |
| AST (UI/) | 5049 (±5398) | 7070 (±5702) | *0.09* |  |
| Factor V (%) | 15 (±10) | 20 (±19) | *0.13* |  |
| INR | 6.3 (±2.1) | 4.6 (±2.0) | *<.001* |  |
| HCO_3-_ (mmol/l) | 19.0 (±7.1) | 18.6 (±5.1) | *0.76* |  |
| Lactate (mmol/l) | 7.9 (±6.8) | 7.1 (±5.6) | *0.53* |  |
| pH | 7.32 (±0.14) | 7.32 (±0.11) | *0.42* |  |
| MELD score | 43 (±8) | 34 (±11) | *<.001* |  |
| *All data are presented as mean (±standard deviation)*  *ALT Alanine aminotransferase, AST: Aspartate aminotransferase, INR : international normalized ratio, PT : prothrombin time, FV: factor V, MELD: model for end stage liver disease* | | | | |

**Supplementary Table S3. Organ support therapies during ICU stay**

|  | **Transplanted (n= 40)** | **Not Transplanted (n=78)** | ***p-value*** |  |
| --- | --- | --- | --- | --- |
| Mechanical ventilation | 17 (43) | 44 (56) | *0.18* |  |
| Neurological indication | 15 (88) | 34 (77) | *0.56* |  |
| Respiratory indication | 2 (12) | 7 (16) | *0.72* |  |
| Renal replacement therapy | 15 (38) | 16 (21) | *0.08* |  |
| MARS therapy | 2 (5) | 7 (9) | *0.72* |  |
| Use of vasopressors | 14 (35) | 31 (40) | *0.76* |  |
| *All data are presented as number (percentage)* | | | | |

**Supplementary Table S4. Characteristic of all deceased patients in ICU and at 1 year discharge**

| **Patient deceased** | **Age** | **Listed** | **LT** | **Etiology** | ***Died in ICU*** | ***Died at 1 year*** | ***Cause of death*** | ***CV*** | ***KCH*** |  |
| --- | --- | --- | --- | --- | --- | --- | --- | --- | --- | --- |
| Patient 1 | 55 | Yes | Yes | HSV | 1 |  | Cardiogenic shock | Yes | Yes |  |
| Patient 2 | 58 | No | No | Ischaemic | 1 |  | MOFS | No | Yes |  |
| Patient 3 | 76 | No | No | Ischaemic | 1 |  | MOFS | No | Yes |  |
| Patient 4 | 50 | Yes | Yes | HVB | 1 |  | MOFS | No | Yes |  |
| Patient 5 | 62 | No | No | Drug induced | 1 |  | MOFS | No | Yes |  |
| Patient 6 | 29 | Yes | Yes | Acetaminophen | 1 |  | Aspergillus pericarditis | Yes | Yes |  |
| Patient 7 | 50 | Yes | Yes | HSV | 1 |  | MOFS | Yes | No |  |
| Patient 8 | 36 | Yes | Yes | Acetaminophen | 1 |  | Hypoxic cardiac arrest | Yes | Yes |  |
| Patient 9 | 57 | No | No | HSV | 1 |  | MOFS | No | Yes |  |
| Patient 10 | 30 | Yes | No | Heatstroke | 1 |  | MOFS | Yes | Yes |  |
| Patient 11 | 33 | No | No | Acetaminophen | 1 |  | Myocardial infarction | No | Yes |  |
| Patient 12 | 63 | Yes | Yes | Drug induced |  | 1 | Hemorrhagic shock post ERCP | No | Yes |  |
| Patient 13 | 77 | No | No | Ischaemic |  | 1 | Unknown cause | No | Yes |  |
| Patient 14 | 58 | No | No | Ischaemic |  | 1 | Esophageal cancer | No | Yes |  |
| Patient 15 | 48 | No | No | Acetaminophen |  | 1 | Tongue cancer | No | Yes |  |
| Patient 16 | 45 | No | No | Acetaminophen |  | 1 | Unknown cause | No | Yes |  |
| Patient 17 | 17 | No | No | Ischaemic |  | 1 | Unknown cause | Yes | Yes |  |
| Patient 18 | 65 | No | No | Drud induced |  | 1 | Unknown cause | Yes | Yes |  |
| Patient 19 | 83 | No | No | Ischaemic |  | 1 | Septic shock | Yes | No |  |
| Patient 20 | 74 | No | No | Acetaminophen |  | 1 | Suicide | Yes | Yes |  |
| Patient 21 | 64 | No | No | Viral |  | 1 | Unknown cause | No | Yes |  |
| *Abbreviations: CV: Clichy Villejuif criteria; KCH: King college criteria; LT: liver transplantation, HSV : herpes simplex virus, MOFS : multiple organ failure syndrome, ERCP : Endoscopic Retrograde Cholangiopancreatography*  *Among patients with an unknown cause of death, all five patients had moved to a different region; their dates of death were retrieved from a national French registry.* | | | | | | | | | | |

**Supplementary Table S5. Era analysis among eligible patients.**

| Era | Eligible | Listed for LT | Transplanted | 1-year survival | Non-transplanted | 1-year transplant-free survival among non-transplanted |
| --- | --- | --- | --- | --- | --- | --- |
| 2000-2010 | 38 | 14/38 (36.8) | 14/38 (36.8) | 30/38 (78.9) | 24 | 18/24 (75.0) |
| 2011-2018 | 38 | 15/38 (39.5) | 14/38 (36.8) | 33/38 (86.8) | 24 | 21/24 (87.5) |
| 2019–2025 | 42 | 14/42 (33.3) | 12/42 (28.6) | 34/42 (81.0) | 30 | 24/30 (80.0) |
| p for trend |  | 0.735 | 0.428 | 0.835 |  | 0.689 |
| *Era intervals were predefined to include approximately equal numbers of patients.*  *P-values for trend were calculated using the Cochran–Armitage trend test.*  *Transplant-free survival was calculated among non-transplanted patients only.* | | | | | | |

**Supplementary Table S6. Baseline characteristics and organ support according to study era among eligible patients.**

| **Variable** | 2000–2010 (n=38) | 2011–2018 (n=38) | 2019–2025 (n=42) | p value |
| --- | --- | --- | --- | --- |
| Age, years | 47.3 ± 15.8 | 45.2 ± 14.0 | 48.6 ± 16.7 | 0.618 |
| Acetaminophen-related ALI | 12 (31.6%) | 19 (50.0%) | 14 (33.3%) | 0.213 |
| Ischemic ALI | 9 (23.7%) | 5 (13.2%) | 6 (14.3%) | 0.401 |
| HE grade 3–4 at admission | 14 (36.8%) | 24 (63.2%) | 25 (59.5%) | 0.041 |
| Total bilirubin at admission (µmol/L) | 199.6 ± 228.9 | 119.5 ± 140.1 | 121.1 ± 141.3 | 0.122 |
| Prothrombin time at admission (%) | 22.2 ± 19.4 | 23.1 ± 14.4 | 25.9 ± 15.7 | 0.596 |
| Creatinine at admission (µmol/L) | 157.9 ± 134.4 | 148.1 ± 137.2 | 133.1 ± 105.3 | 0.767 |
| Mechanical ventilation at admission | 8 (21.1%) | 14 (36.8%) | 11 (26.2%) | 0.297 |
| Vasopressors at admission | 12 (31.6%) | 14 (36.8%) | \| 19 (45.2%) \| \| --- \| | 0.420 |
| Renal replacement therapy at admission | 0 (0%) | 3 (7.9%) | 1 (2.4%) | 0.159 |
| MARS therapy during ICU stay | 2 (5.3%) | 2 (5.3%) | 5 (11.9%) | 0.437 |

*Continuous variables are expressed as mean ± SD and compared using one-way ANOVA.
Categorical variables are expressed as n (%) and compared using χ² or Fisher’s exact test.***Supplementary figure S1.** Evolution of encephalopathy grade between admission (baseline) and the worst encephalopathy grade during the ICU stay in transplanted (A) and not transplanted patients (B).

**
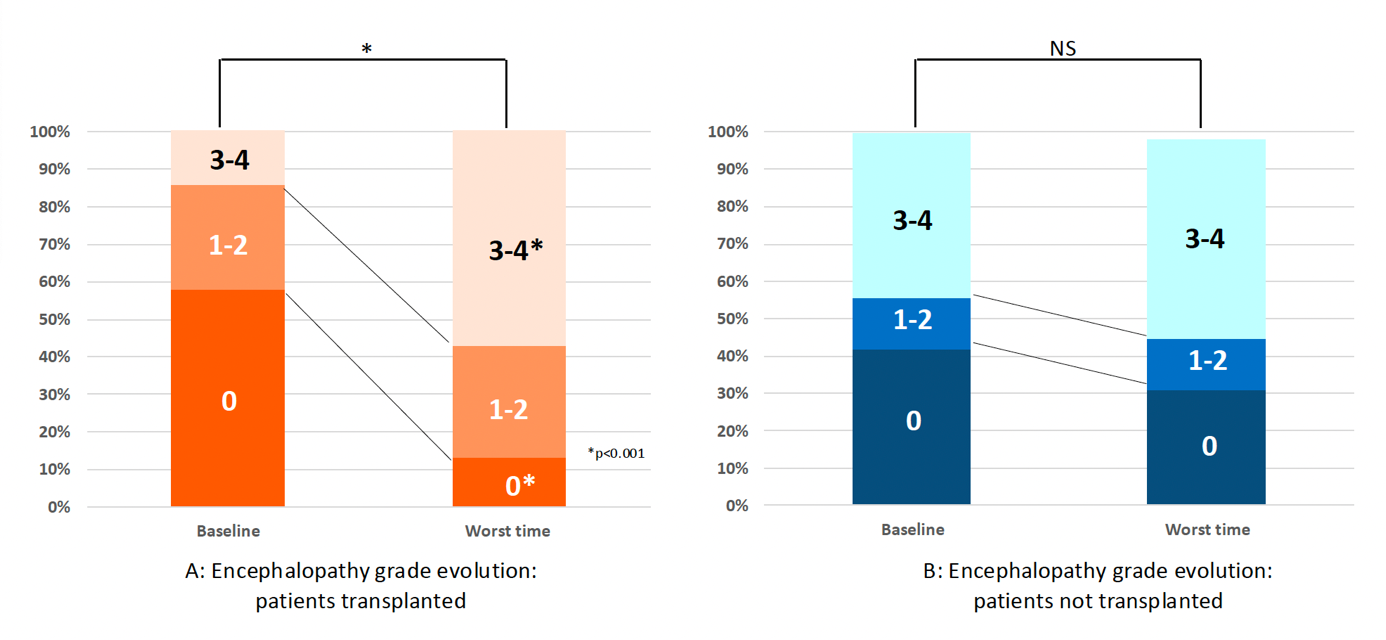
**


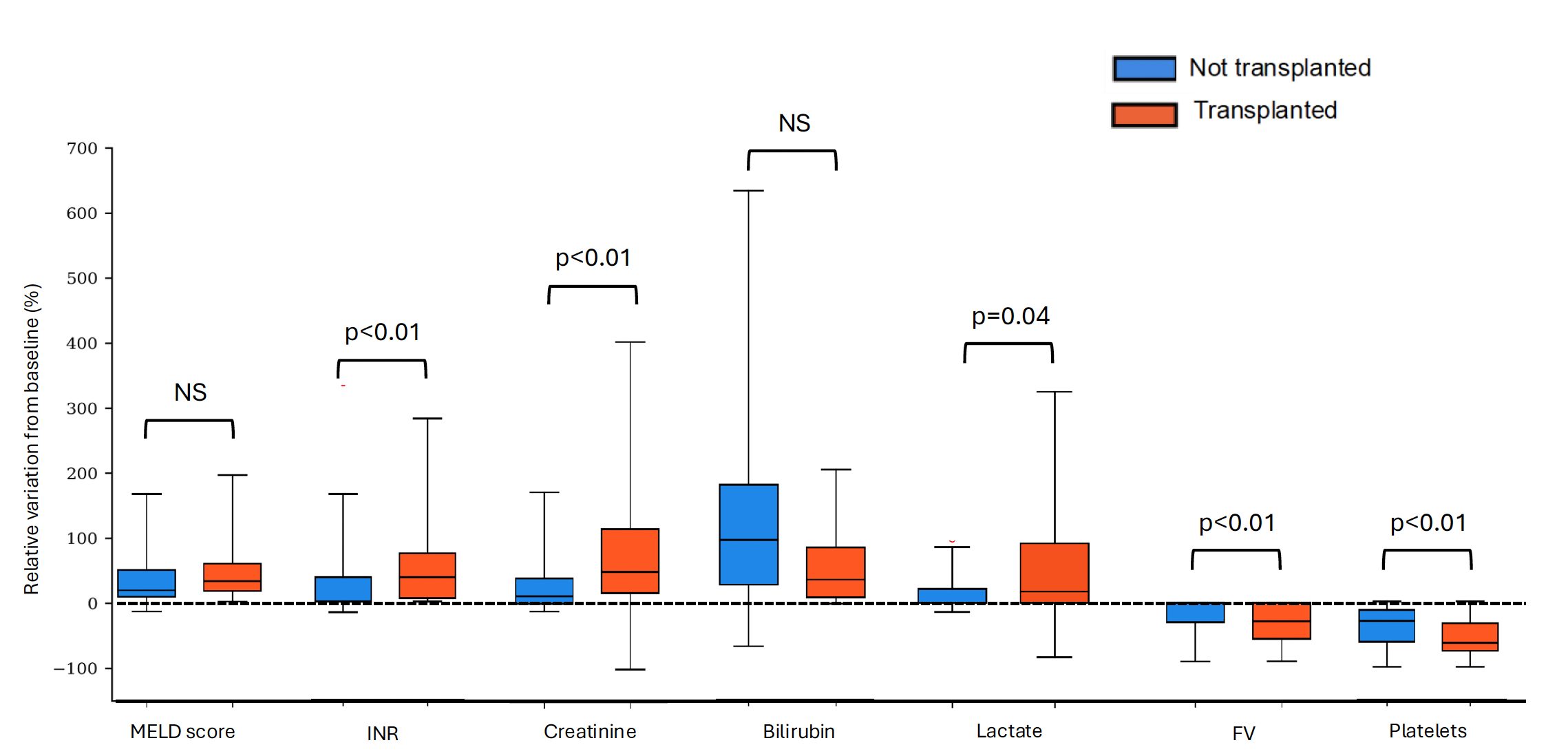
**Supplementary figure S2.** Relative variation from baseline to worst values of main biological variables and comparison between transplanted and not transplanted groups
